# Supplementary material for: NDAT Targets PI3K-Mediated PD-L1 Upregulation to Reduce Proliferation in Gefitinib-Resistant Colorectal Cancer
Source: Cells. 2020 Aug 3;9(8):1830. doi: 10.3390/cells9081830 (PMC7464180; doi:10.3390/cells9081830)
Supplement: Supplementary file 1 [file cells-09-01830-s001.pdf]

Supplementary data

Specimen preparation and tumor cell isolation

The isolation of and culture procedures for primary cultures of human CRC cells were modified from previous studies [1, 2]. Briefly, after resection, intestinal tissue was washed with phosphate-buffered saline (PBS) and then placed in a sterile container containing a transport buffer. The specimens were supplemented with 5× penicillin-streptomycin-amphotericin B (500 U/mL, 500 μg/mL, and 12.5 μg/mL, respectively) (Sigma-Aldrich Corp., St. Louis, MO, USA) and then transported to the laboratory. After removal of excess fat and normal tissue, samples were washed with PBS (supplemented with 5× penicillin-streptomycin-amphotericin B) and then reduced to 0.5-2.0 mm^3 pieces with a medical scalpel and scissors. Tumor fragments were incubated with pre-warmed digestion medium (RPMI 1640 medium supplemented with 1.5 mg/mL collagenase type I and 20 μg/mL hyaluronidase) (Sigma-Aldrich) for 3 h in an incubator at 37°C. The cell suspension was filtered with cell strainer (mesh size: 70 μm) (Thermo Scientific, Rockford, IL, USA) and washed with RPMI 1640 supplemented with 10% fetal bovine serum (FBS). The samples were incubated with ammonium chloride solution-Tris solution (0.16 M NH_4Cl and 0.17 M Tris, pH 7.2) to remove erythrocyte contamination. Finally, the recuperated cell population was washed and resuspended in the culture medium. Four primary human CRC cell samples (colo_150624, colo_150812-2, colo_160224 and colo_160426) were isolated and cultured in RPMI 1640 medium with 10% FBS and antibiotics (penicillin 100 IU/mL, streptomycin 100 μg/mL, amphotericin B 2.5 μg/mL) until use. Before these treatments, cells were placed in serum-free medium for 24 h starvation.

Xenografts

Forty nude mice (BALB/cAnN.Cg-Foxn1nu/CrlNarl, male) were purchased from National Laboratory Animal Center (Taipei, Taiwan) and were housed in a reserved, pathogen-free facility and were treated by the protocols approved by the Institutional Animal Care and Use Committee of the National Defense Medical Center, Taipei, Taiwan (IACUC-15-340). As described previously [3], mice were anesthetized with xylazine (10 mg/kg). Each mouse was subcutaneously inoculated with aliquots of HCT116 cells (1×10^6 cells/100 μl Matrigel) (BD Matrigel™ Basement Membrane Matrix, BD Biosciences, San Jose, CA, USA) on each dorsal side using a 26-gauge needle on a tuberculin syringe. After inoculation, the animals were further intraperitoneally treated with solvent (PBS, 8 mice), gefitinib (in PBS with 0.5% Tween 80, 10 mg/kg, twice a week, 8 mice), NDAT (in PBS, 0.05 mg/kg, twice a week, 4 mice; 0.1 mg/kg, twice a week, 4 mice; 1 mg/kg, twice a week, 8 mice), or the combination of gefitinib (10 mg/kg) and NDAT (1 mg/kg) (8 mice) for 5 weeks. The volume of tumors was measured twice a week. The size of tumors was measured by using digital calipers, and the volume was calculated as (length × width × width)/2 and expressed as cubic millimeters (mm^3) [4]. The fold change of tumor volume was calculated from the final measured volume of tumors divided by the first measured volume of tumors. These results were expressed as mean ± SD. After 5 weeks of' drug treatment, all animals were sacrificed, and the tumor masses were resected and collected. Half of the tumor masses were fixed in 4% paraformaldehyde and paraffin-embedded for routine hematoxylin and eosin stain (H&E stain) and immunohistochemical staining; the other half of tumor masses were put in liquid nitrogen and then stored at -80°C for further qPCR examinations.

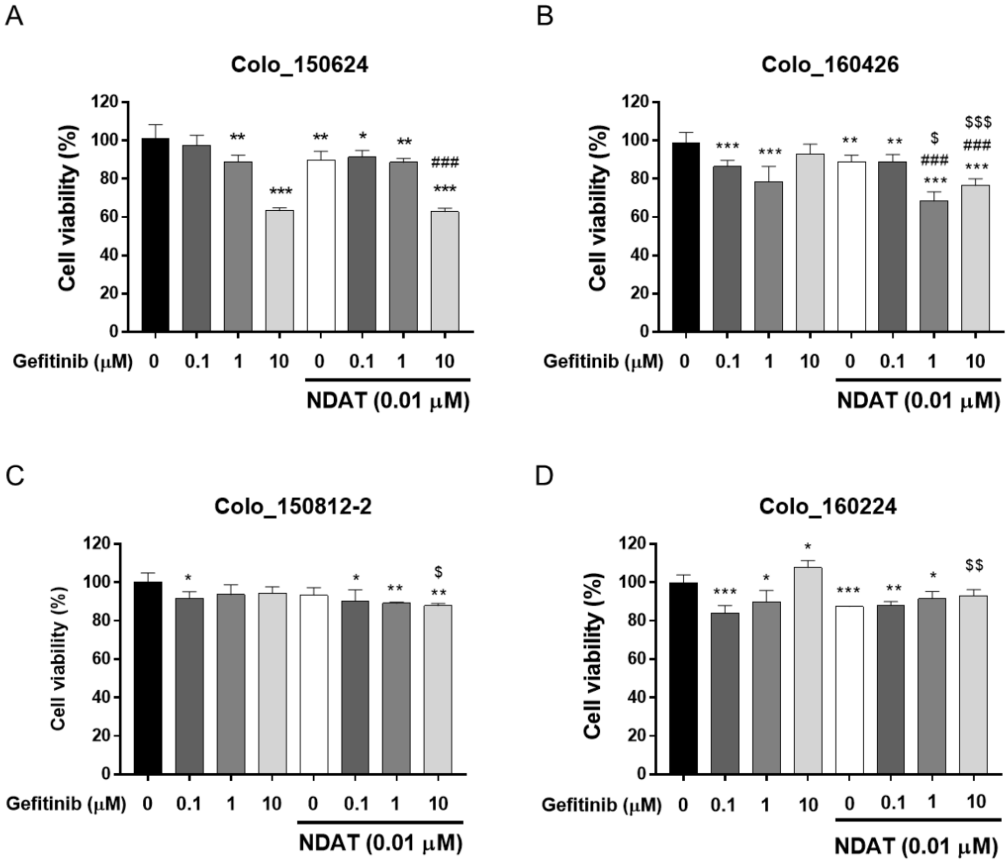

Figure S1. NDAT augments gefitinib-stimulated anti-proliferation in primary cultures of human CRC cells. Four established primary human CRC cell cultures, Colo_150624 (A), Colo_160426 (B), Colo_150812-2 (C), and Colo_160224 (D), were seeded in 96-well plates and treated with different concentrations of gefitinib (0.1, 1 and 10 μM), NDAT (0.01 μM), or their combination. Media with drugs were refreshed daily for 6 days. Cell viability was examined with MTT assay. N = 6. Data are expressed as mean ± SD; * $p < 0.05$, ** $p < 0.01$, *** $p < 0.001$, compared with untreated control; # $p < 0.05$, ## $p < 0.01$, ### $p < 0.001$, compared with NDAT; $ $p < 0.05$, $$ $p < 0.01$, $$$ $p < 0.001$, compared with gefitinib.

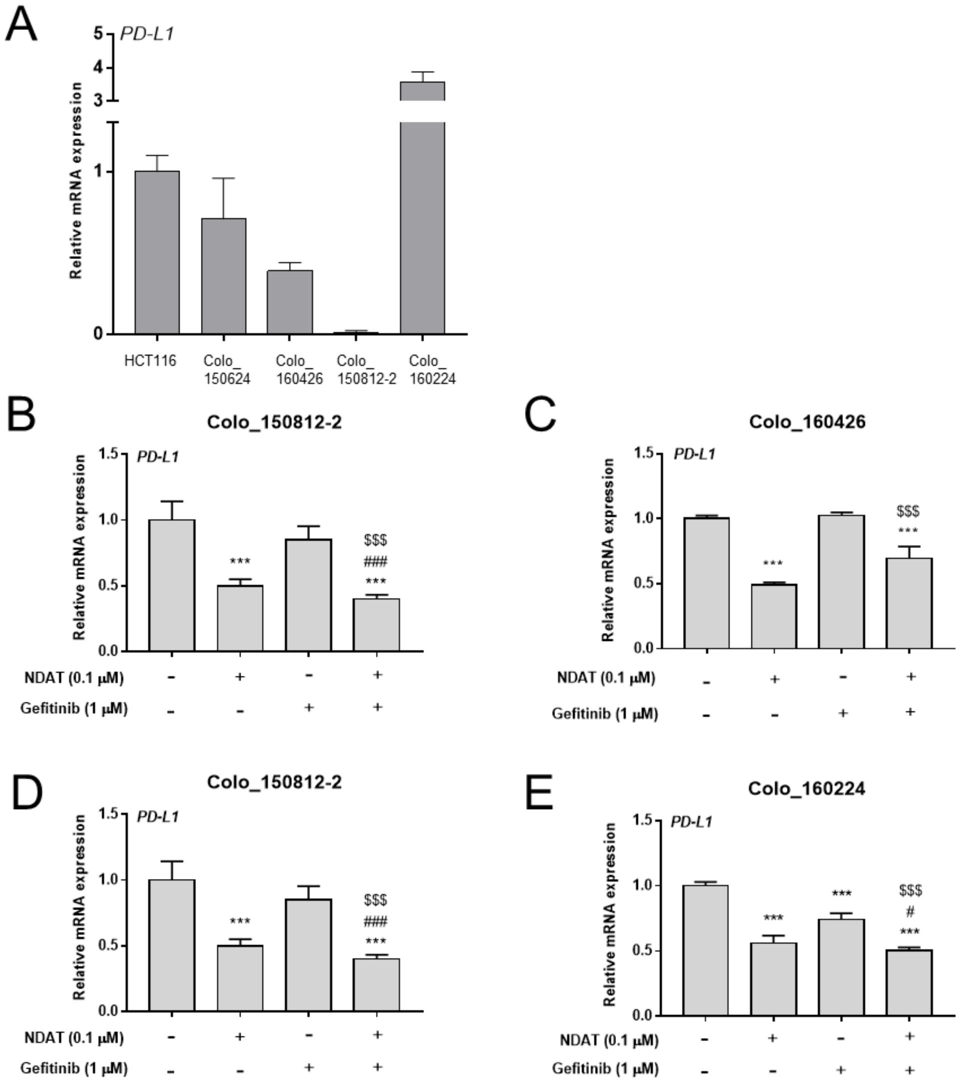

Figure S2. NDAT attenuate expression of PD-L1 regulated by gefitinib in human CRC primary cell cultures. (A) Basal mRNA expression of PD-L1 among HCT116 cells and four established primary human CRC cell cultures was evaluated by qPCR. N = 3 (Data were expressed as mean ± SD. A-E: the subsets after post hoc analysis of the significant differences were obtained by one-way ANOVA). Four established primary human CRC cell cultures, Colo_150624 (B), Colo_160426 (C), Colo_150812-2 (D), and Colo_160224 (E), were seeded in 6-well plates and treated with different concentrations of gefitinib (1 μM), NDAT (0.1 μM), or their combination after starvation for 24 h. Cells were harvested and total RNA was extracted. qPCR was conducted for PD-L1 expression. N = 6. Data are expressed as mean ± SD; * $p < 0.05$, *** $p < 0.001$, compared with untreated control; # $p < 0.05$, ## $p < 0.01$, ### $p < 0.001$, compared with NDAT; $$ $p < 0.01$, $$$ $p < 0.001$, compared with gefitinib.

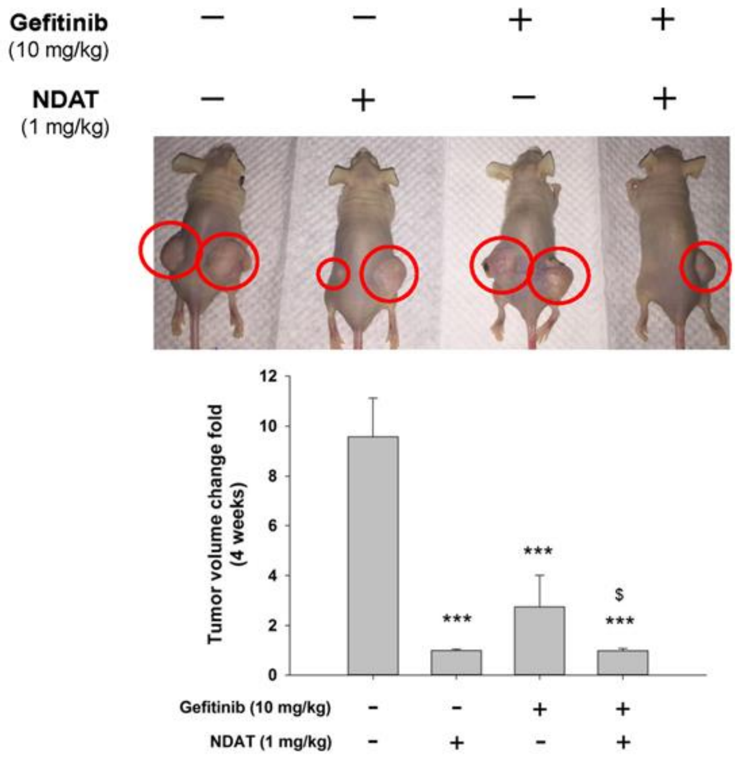

Figure S3. NDAT and gefitinib inhibit tumor growth of HCT116 xenograft. Nude mide were inoculated with HCT116 subcutanenouly and treated intraperitoneally with NDAT, gefitinib or in combination for five weeks. The fold change of tumor volume was calculated from the final measured volume of tumors divided by the first measured volume of tumors. These results were expressed as mean ± SD; *** $p < 0.001$, compared with untreated control; $ $p < 0.05$, compared with gefitinib.

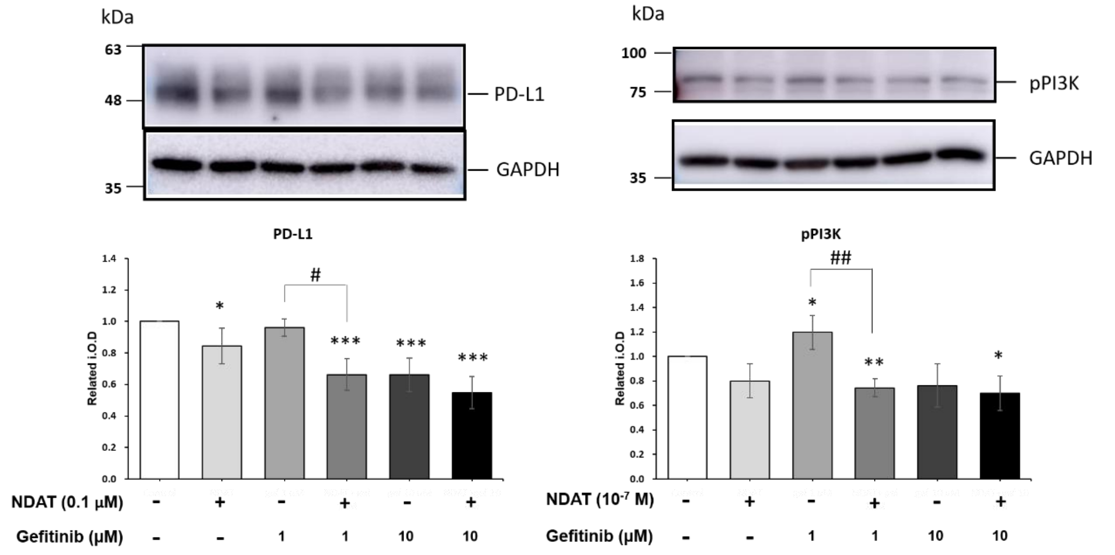

Figure S4. **NDAT inhibited PD-L1 accumulation in Colo_160224.** Primary cell culture was harvested and extracted. Western blotting analyses of total protein were conducted for PD-L1. Number of independent experiments (N) = 4. Data are expressed as mean ± SD; * $p < 0.05$, *** $p < 0.001$, compared with untreated control; ## $p < 0.01$, compared with NDAT; $ $p < 0.05$, compared with gefitinib.

References:

1. Failli A, Consolini R, Legitimo A, et al. The challenge of culturing human colorectal tumor cells: the establishment of a cell culture model by the comparison of different methodological approaches. *Tumori* 2009; 95: 343-347. 2009/08/20.
2. Ali MY, Anand SV, Tangella K, et al. Isolation of Primary Human Colon Tumor Cells from Surgical Tissues and Culturing Them Directly on Soft Elastic Substrates for Traction Cytometry. *J Vis Exp* 2015: e52532. 2015/06/13. DOI: 10.3791/52532.
3. Chang TC, Chin YT, Nana AW, et al. Enhancement by Nano-Diamino-Tetrac of Antiproliferative Action of Gefitinib on Colorectal Cancer Cells: Mediation by EGFR Sialylation and PI3K Activation. *Hormones & cancer* 2018 2018/09/07. DOI: 10.1007/s12672-018-0341-x.
4. Tomayko MM and Reynolds CP. Determination of subcutaneous tumor size in athymic (nude) mice. *Cancer chemotherapy and pharmacology* 1989; 24: 148-154. Comparative Study
Research Support, U.S. Gov't, P.H.S. 1989/01/01.
